# Combining Metagenomic Sequencing With Whole Exome Sequencing to Optimize Clinical Strategies in Neonates With a Suspected Central Nervous System Infection

**DOI:** 10.3389/fcimb.2021.671109

**Published:** 2021-06-18

**Authors:** Mengmeng Ge, Mingyu Gan, Kai Yan, Feifan Xiao, Lin Yang, Bingbing Wu, Mili Xiao, Yin Ba, Rong Zhang, Jin Wang, Guoqiang Cheng, Laishuan Wang, Yun Cao, Wenhao Zhou, Liyuan Hu

**Affiliations:** ^1^ Department of Neonatology, Children’s Hospital, Fudan University, Shanghai, China; ^2^ Clinical Genetic Center, Children’s Hospital of Fudan University, Shanghai, China; ^3^ Children’s Hospital of Fudan University, Shanghai Key Laboratory of Birth Defects, The Translational Medicine Center of Children Development and Disease of Fudan University, Shanghai, China

**Keywords:** metagenomic sequencing, WES, suspected central nervous system infection, neonates, NICU

## Abstract

**Objectives:**

Central nervous system (CNS) infection has a high incidence and mortality in neonates, but conventional tests are time-consuming and have a low sensitivity. Some rare genetic diseases may have some similar clinical manifestations as CNS infection. Therefore, we aimed to evaluate the performance of metagenomic next-generation sequencing (mNGS) in diagnosing neonatal CNS infection and to explore the etiology of neonatal suspected CNS infection by combining mNGS with whole exome sequencing (WES).

**Methods:**

We prospectively enrolled neonates with a suspected CNS infection who were admitted to the neonatal intensive care unit(NICU) from September 1, 2019, to May 31, 2020. Cerebrospinal fluid (CSF) samples collected from all patients were tested by using conventional methods and mNGS. For patients with a confirmed CNS infection and patients with an unclear clinical diagnosis, WES was performed on blood samples.

**Results:**

Eighty-eight neonatal patients were enrolled, and 101 CSF samples were collected. Fourty-three blood samples were collected for WES. mNGS showed a sample diagnostic yield of 19.8% (20/101) compared to 4.95% (5/101) for the conventional methods. In the empirical treatment group, the detection rate of mNGS was significantly higher than that of conventional methods [27% vs. 6.3%, p=0.002]. Among the 88 patients, 15 patients were etiologically diagnosed by mNGS alone, five patients were etiologically identified by WES alone, and one patient was diagnosed by both mNGS and WES. Twelve of 13 diagnoses based solely on mNGS had a likely clinical effect. Six patients diagnosed by WES also experienced clinical effect.

**Conclusions:**

For patients with a suspected CNS infections, mNGS combined with WES might significantly improve the diagnostic rate of the etiology and effectively guide clinical strategies.

## Introduction

Central nervous system infections (CNS infections), including meningitis, encephalitis, and abscesses, refer to inflammation of the brain and spinal cord caused by various pathogenic microbes. For neonates with a CNS infection, early identification of the pathogen is the key to accurate treatment. Because of their immature immune function, newborns are at high risk of all types of infections. (Yu et al., 2018)Additionally, their blood-brain barrier (BBB) is immature, and thus microorganisms and cytokines can easily cause CNS infection by passing through the BBB. The incidence of neonatal meningitis is 0.3/1,000-6.1/1,000 live births, varying by geographic location. (Furyk et al., 2011; Heath et al., 2011) The mortality rate of neonatal meningitis is 10% - 25%, and the incidence of long-term sequelae is 23-38%. (Di Mauro et al., 2019; El-Naggar et al., 2019)Culture, the gold standard of clinical diagnosis, is time consuming and has low sensitivity. Notably, 60% - 85% of the bacteria detected by using the metagenomics method do not grow on standard bacterial culture media. (Simner et al., 2018) Polymerase chain reaction (PCR)-based and antigen or antibody methods only detect a limited number of pathogens in a single experiment. A presumption of pathogens is needed to select the appropriate primers and probes. (Marcilla-Vazquez et al., 2018; Whiteduck-Léveillée et al., 2016) The diagnostic workup for many patients requires extensive and serial testing that utilizes a combination of culture, antigen, serologic, and molecular methods, resulting in delayed or missed diagnoses and increased costs.

Metagenomic next-generation sequencing (mNGS) represents a comprehensive method by which almost all potential pathogens, including viruses, bacteria, fungi and parasites, can be accurately identified in a single measurement. (Simner et al., 2018)mNGS has developed rapidly and is characterized by a short turnaround time, unbiased detection and semiquantitative value in pathogen detection. To date, several studies have confirmed the clinical application value of mNGS in the diagnosis and treatment guidance of various infectious diseases, including CNS infections, bloodstream infections, respiratory infections, focal infections, etc. (Wilson et al., 2019; Zhang et al., 2019) However, little applied research has been conducted in the neonatal population.

Although microbiological investigations have improved significantly in recent decades, the etiological agents of acute meningoencephalitis remain unknown in approximately 50% of cases. (Granerod et al., 2010) CNS infection involving deep viscera occurs in the neonatal period, and the possibility of a congenital immune deficiency must be excluded. Some rare genetic diseases may have similar clinical manifestations as CNS infections, such as seizures, coma, and dystonia, which may be due to rare genetic diseases. (Sun et al., 2020) Newborns are at high risk of rare diseases, and early whole-exome sequencing (WES) tests can facilitate a genetic diagnosis and guide clinical management in critically ill neonates in the neonatal intensive care unit (NICU). Importantly, for affected families, a better understanding of the genetic basis of rare disease translates to a more accurate prognosis, management, surveillance and genetic advice; stimulates research into new therapies; and enables the provision of better support.

In 2017, France recommended mNGS as level 1 evidence in the Guidelines for the Treatment of Adult Infectious Encephalitis to assist in the clinical management of central nervous system infection. (Stahl et al., 2017) However, published reports describing the usefulness of mNGS in patients with CNS infections were limited to individual patients or small retrospective case series. (Wilson et al., 2014; Greninger et al., 2015) Usually, the cases were derived from adults or older children. (Wilson et al., 2018) Therefore, we conducted a prospective study in Shanghai, China, to evaluate the performance of mNGS combined with WES genetic tests in the diagnosis of neonatal suspected CNS infection.

## Methods

### Ethical Approval

The study was conducted in accordance with the Declaration of Helsinki. The study was approved by the Research Ethics Committee of the Children’s Hospital of Fudan University (NO.2019-300). The trial was registered in the Clinical Trial Registry (https://clinicaltrials.gov/; NCT04320810).

### Study Design

**Figure 1:**
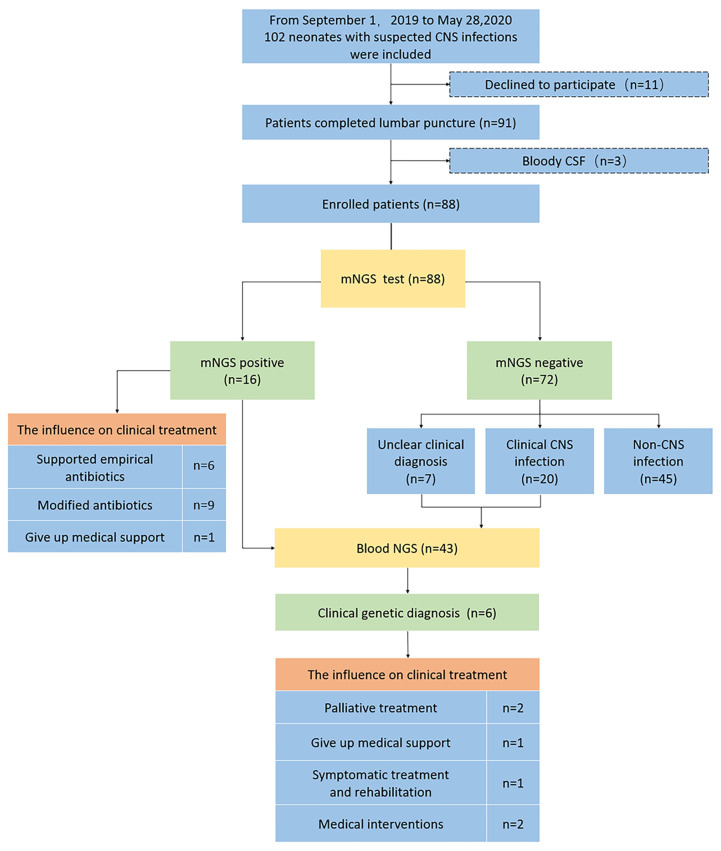
Flowchart of the study. CNS central nervous system, mNGS metagenomic next-generation sequencing, NGS next-generation sequencing, CSF cerebrospinal fluid. Non-CNS infection included neonatal sepsis/clinical sepsis, pneumonia, urinary tract infections, hypoxic ischemic encephalopathy, hydrocephalus, and intracranial hemorrhage with signs of infection. Unclear clinical diagnosis included encephalopathy with signs of infection. CSF pleocytosis was defined as a CSF white blood cells (WBC) count ≥20/mm^3^ (0–28 days of age) or >5/mm^3^ (older children). Definite meningitis was defined as (1) the presence of a pathogen in the CSF or (2) CSF pleocytosis with either a relevant pathogen present in the blood or an alternative positive diagnostic test. Probable meningitis was defined as CSF pleocytosis with a relevant pathogen detected at a sterile site (except blood) and a discharge diagnosis of meningitis. Aseptic meningitis of unknown etiology was defined as CSF pleocytosis without a pathogen identified. Possible meningitis was defined as meningitis as the discharge diagnosis without microbiological confirmation. In this study, the etiologic diagnosis was definite meningitis, and the clinical diagnosis included probable meningitis, aseptic meningitis of unknown an etiology and possible meningitis.

We enrolled all neonates suspected of having CNS infection between September 1, 2019, and May 31, 2020, who were hospitalized in the NICU, Children’s Hospital of Fudan University in Shanghai, China. We conducted a single-center prospective study to evaluate the performance of mNGS in diagnosing neonatal CNS infections (**Figure 1**). The inclusion criteria were all clinically stable neonates suspected of having CNS infection. (Ku et al., 2015) The exclusion criteria were parental refusal of a lumbar puncture or bloody cerebrospinal fluid (CSF). CSF must be sent for mNGS and conventional tests at the same time, which include routine CSF and biochemical tests, culture, smear, and HSV (herpes simplex virus)-PCR. An orthogonal confirmation test was conducted for positive testing of pathogens only identified using mNGS (Detailed in **Supplementary Materials**).

CSF pleocytosis was defined as CSF white blood cells (WBC) counts ≥20/mm3 (0–28 days of age) or >5/mm3 (older children). Definite meningitis was defined as (1) the presence of a pathogen in the CSF or (2) CSF pleocytosis with either a relevant pathogen present in the blood or an alternative positive diagnostic test. Probable meningitis was CSF pleocytosis with a relevant pathogen detected at a sterile site (except blood) and a discharge diagnosis of meningitis. Aseptic meningitis of unknown etiology was defined as CSF pleocytosis without a pathogen identified. Possible meningitis was defined as meningitis as the discharge diagnosis without microbiological confirmation. (Ramasamy et al., 2018) In this study, the etiologic diagnosis was definite meningitis, and the clinical diagnosis included probable meningitis, aseptic meningitis of unknown etiology and possible meningitis. For all patients with confirmed CNS infection (including clinical diagnosis and etiologic diagnosis) and patients with an unclear clinical diagnosis, WES was performed to clarify whether diseases with similar CNS infection symptoms might be caused by other gene defects to guide clinical intervention and strategies.

We calculated the sensitivity, specificity, positive predictive value (PPV) and negative predictive value (NPV) of mNGS compared with conventional testing results to evaluate the overall diagnostic performance of mNGS. Based on the medical history before sample isolation, the subjects were divided into an empirical antibiotic treatment group and a nontreatment group, and the detection rates of mNGS and conventional methods in these two groups were calculated.

We collected data on history, birth weight, gestational age, sex, delivery method, age, evidence of lumbar puncture, peripheral blood count (CBC), serum cytomegalovirus PCR, blood and CSF culture results, CSF parameters, CSF HSV-PCR, CSF CMV-PCR and NGS results. The method of mycobacteria culture is different from that of general bacteria culture. However, since the patients enrolled in our study didn’t have a high risk of mycobacteria infection, the culture in the study only included general bacteria culture. The culture methods are detailed in **Supplementary Materials**.

## mNGS and Data Analysis

DNA was extracted from 1 ml of CSF using a QIAamp UCP Pathogen Mini Kit (Qiagen, Hilden, Germany). The DNA sequencing library was constructed using KAPA Hyper Prep Kits (Roche, Basel, Switzerland). DNA was sequenced on an Illumina NovaSeq machine using a 150-cycle paired-end sequencing kit. At least 30 million paired-end reads were obtained for each sample. Approximately 2 hours were required for DNA extraction, 4 hours for library construction, and 44 hours for sequencing. A nontemplate control (NTC) was processed with samples in parallel, from DNA extraction to sequencing.

Adaptors and low-quality reads were trimmed using Trimmomatic v0.39 (20). Trimmed reads were aligned to a human reference genome (GRCh38) using Bowtie v2.3.4. After removing human reads, the remaining reads were aligned with the microorganism reference genome database using Centrifuge v1.0.3. Reads that aligned to multiple different species and reads with alignment rates lower than 70% were filtered.

We developed an NTC-based strategy to filter reagents and laboratory contamination. Sequencing reads of microbes were standardized as the RPM value (the number of microbe reads per million nonhuman sequenced reads). If a microbe was detected in the NTC, an RPM-ratio (RPM-Sample/RPM-NTC) of 10 was used as the cutoff. If a microbe was not detected in the NTC, an RPM sample of 3 was used as the cutoff for bacteria, fungi, and parasites; for viruses, >3 nonoverlapping reads were used as the cutoff.

Strains detected by using mNGS were validated with a probe-based qPCR method. Probes and primers targeting each species detected by using mNGS were ordered from ThermoFisher ScientificTaqMan Gene Expression Assays (Thermo Fisher Scientific, San Francisco, CA,USA). Assay IDs and targeted species are listed in **Supplementary Table 1**. All qPCR assays were performed on a StepOnePlus instrument (Applied Biosystems, Carlsbad, CA). The mix consisted of 2.5 µl of 4× TaqMan Fast Virus 1-Step Master Mix, 0.5 µl of 20× TaqMan Gene Expression Assay, 1 µl of DNA templates, and 6 µl of nuclease-free water. The qPCR conditions were as follows: preincubation at 95°C for 10 min, amplification for 40 cycles at 95°C for 15 s and 60°C for 1 min.

### Genetic Tests Using WES and Data Analysis

Genomic DNA of the patients was extracted from whole blood using the QIAamp DNA Blood Mini Kit (Qiagen, Hilden, Germany). DNA preparation and high-throughput sequencing were performed using standard protocols by Wuxi NEXTCODE in a sequencing laboratory compliant with Clinical Laboratory Improvement Amendments (CLIA; 288 Fute Zhong Road, Waigaoqiao Free Trade Zone Shanghai 200131, China, CLIA ID 99D2064856).

Low-quality reads were discarded from the raw data to generate clean reads, which were aligned to the reference human genome (UCSC hg19) with Burrows-Wheeler Aligner (BWA; v.0.5.9-r16). Procedures for variant calling were performed according to the GATK best practice (V.3.2). We used ANNOVAR and VEP software to annotate SNVs. The allelic frequencies were annotated from the public datasets from the 1000 Genomes, Exome Sequencing Project (EVS6500) and Exome Aggregation Consortium (ExAC), Kyoto, and Dutch allelic frequency databases. The interpretation of sequence variants followed the published pipeline established in house, according to the American College of Medical Genetics guidelines. (Richards et al., 2015; Yang et al., 2019) Variants were confirmed by Sanger sequencing. PCR products were sequenced using the BigDye Terminator v3.1 Cycle Sequencing Kit (Applied Biosystems, Carlsbad, CA) with an ABI 3730 Genetic Analyzer. Mutation Surveyor Software (Softgenetics, State College, PA) was used for data analysis.

### Statistical Analysis

Continuous variables with normal distributions are presented as the means ± standard deviations and as medians and interquartile ranges (IQRs) when the data had a nonnormal distribution. Categorical variables are presented as percentages. Fisher’s exact test was used to evaluate independent binomial variables. The analysis was performed using IBM SPSS statistics 23.

## Results

### General Characteristics

One hundred two patients were screened for review and prospective enrollment in this study. The parents of eleven patients refused a lumbar puncture, and three patients had bloody CSF; all fourteen were subsequently excluded. Eighty-eight patients were enrolled, and 101 CSF samples were collected (**Figure 1**). A total of 44.3% of the patients (39/88) were delivered by cesarean section. The median age at admission (54.5% were male) was 10 days (31 days), the median gestational age was 38 + ^1^ weeks (6 weeks), and the birth weight was 3000g (1449g). (**Table 1**).

**Table 1:** Baselines characteristics of participants*.

Characteristic	Clinical value
Gestational age (median; IQR; weeks)	38+1(6)
Age (median; IQR; d)	10(31)
Birth weight (median; IQR; g)	3000(1449)
Sex	
Male	54.5% (48/88)
Female	45.5% (40/88)
Delivery route	
Vaginal delivery	55.7% (49/88)
Cesarean section	44.3% (39/88)
Evidence of lumbar puncture	
Fever	25% (22/88)
Sepsis/clinical sepsis	34.1% (30/88)
Follow-up examination	22.7% (20/88)
Seizure	12.5% (11/88)
Coma	1.1% (1/88)
Hydrocephalus	1.1% (1/88)
Intracranial hemorrhage	2.3% (2/88)
Dystonia	1.1% (1/88)
Medication	
Empirical treatment	62.4% (63/101)
Nontreatment	37.6% (38/101)

*CNS denotes central nervous system, IQR, interquartile range.

### Overall Diagnostic Performance of mNGS

**Figure 2:**
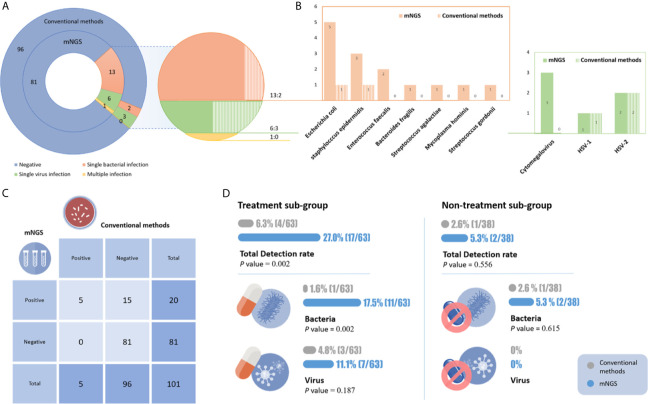
**(A)** The results of mNGS and conventional tests. **(B)** Distribution of pathogens identified by mNGS and conventional tests. **(C)** Comparison of mNGS and conventional methods. **(D)** The detection rate of the different methods in the two subgroups. In the empirical treatment group, the detection rate of mNGS was significantly higher than that of conventional methods, especially for bacterial infections.

Of the 101 CSF samples, 4.95% (5/101) of the samples were positively detected by conventional methods, while 19.8% (20/101) of the samples were positively detected by mNGS (**Figures 2A, B**). Multiple infections were observed in 5% (1/20) of mNGS-positive samples, while conventional methods did not detect these infections. As shown in **Figure 2C**, mNGS showed a 100% (5/5) sensitivity, 84.4% (81/96) specificity, 25% PPV and 100% NPV compared to conventional methods.

mNGS enables much broader pathogen species detection. In 20 positive samples, 11 microbe species were detected by mNGS, including eight bacteria and three viruses. Rare etiological agents (*Mycoplasma hominis*) of CNS infection were observed. Conventional methods only detected four microbe species, including one *Escherichia coli*, one *Staphylococcus epidermidis*, one HSV-1 and two HSV-2. The top three causative pathogens identified were *Escherichia coli*, *Staphylococcus epidermidis* and CMV (**Figures 2A, B**).

The advantages of mNGS were more pronounced in newborns who had already been treated with antibiotics. The samples were divided into two groups. Prior to CSF sampling, 62.4% (63/101) and 37.6% (38/101) of patients received empirical therapy or no empirical therapy, respectively. In the empirical treatment group, the detection rate of mNGS was significantly higher than that of conventional methods [27% (17/63) vs. 6.3% (4/63), p=0.002]. In the nontreatment group, their detection rates were 5.3% (2/38) and 2.6% (1/38), respectively. The rate of bacterial CNS infection detection by mNGS was significantly higher than that of culture in both the empirically treated and nontreated groups (17.5% vs. 1.6%, p=0.002; 5.3% vs. 2.6%, respectively) (**Figure 2D**).

### WES Genetic Test

A combination of CSF mNGS and blood WES was used to identify the etiology of neonates with a suspected CNS infection. According to our diagnostic flowchart, WES was performed in 43 cases. Ultimately, six patients were found to have a genetic etiology using WES. In addition to signs of infection, five of the six patients had seizures or manifested other neurological abnormalities. The mother of patient C4 had a fever before delivery, and the child developed seizures and a fever after birth; however, her CSF tests were all negative, and she was finally confirmed to have seizures that began in the neonatal period due to a mutation in KCNQ3. Patient C43 had severe metabolic acidosis, seizures, and malaise. His CSF tests were also negative, and he was eventually diagnosed with argininosuccinic aciduria. In patient C87, multiple *Streptococcus agalactiae* infections were detected in the CSF by mNGS (before entering our hospital), and CMV in blood and a genetic test confirmed that he had primary immunodeficiency disease (PID) (**Table 2**).

**Table 2:** Six patients with definited genetic diagnoses*.

	mNGS	WES	Variant	Zygosity	Diseases	Outcomes
C87	GBS	*IKZF1*	NM_006060; exon8: c.1393G>C (p.E465Q)	Het	Immunodeficiency, common variable,13	Follow-up in the Immunology Department
	CMV					
C4	–	*KCNQ3*	NM_004519; exon13: c.1768G>A (p.A590T)	Het	Seizures, begin neonatal,2	N
C21	–	*MOCS2*	NM_004531; exon4: c.167_168delA (p. D57Mfs*7)	Hom	Molybdenum cofactor deficiency B	Died
C31	–	*ZMIZ1*	NM_020338; exon10: c.703C>T (p.Q235X)	Het	Neurodevelopmental disorder with dysmorphic facies and distal skeletal anomalies	Nasal tube feeding; High muscle tension
C35	–	*SCN2A*	NM_001371246; exon6: c.662T>C (p.V221A)	Het	Epileptic encephalopathy, earlyinfantile,11	Drug controlled convulsions
C43	–	*ASL*	NM_000048; exon10: c.706C>T (p.R236W)	Het	Argininosuccinic aciduria	Drug controlled convulsions; Limited protein intake

*mNGS denotes metagenomic next-generation sequencing, WES denotes whole- exome sequencing, CMV denotes cytomegalovirus, GBS denotes S.agalactiae, and N denotes normal.

Before patient C87 was referred to our hospital, he had a repeated fever for 40 days. GBS was detected in the CSF and blood mNGS, and penicillin was used for anti-infection treatment. He was referred to our hospital due to his fever and the lack of improvement in his CSF parameters. Follow-up of CSF mNGS was negative, while blood mNGS and PCR indicated CMV, and ganciclovir was added to the treatment.

The mother of patient C4 had a fever before birth, and the child developed seizures and fever after birth, although her CSF tests were all negative.

The mother of C21 had premature rupture of the membrane for 17 hours, and the infant experienced seizures after birth. Her routine blood test suggested that both WBC counts and C-reactive protein (CRP) levels were elevated, while CSF parameters were negative.

Patient C31 was admitted with a fever and increased muscular tension, but his CSF parameters were negative.

Patient C35 was admitted to the hospital with a recurrent fever and seizures for 6 days, and his routine blood and CSF fluid parameters were negative.

Patient C43 had severe metabolic acidosis, seizures, and malaise. His CSF tests were negative.

### The Effect on Clinical Treatment

Diagnosis with mNGS guides the clinical treatment of neonatal CNS infection. Sixteen CNS infections were etiologically diagnosed in 88 patients. Among these 16 patients, three were identified by both mNGS and conventional tests, and 13 (14.8%) were identified by mNGS alone. Twelve of 13 diagnoses made solely based on mNGS had a likely clinical effect (one patient’s parents stopped treatment), with nine of 13 changing antibiotic treatments. The management of clinical treatments based on mNGS is divided into two categories. First, treatment was supported by mNGS results, e.g., in patient C60, the clinician empirically selected meropenem and then continued to use it until the patient was cured according to mNGS. Second, the treatment was modified according to the pathogen identified by mNGS, e.g., ganciclovir was added after mNGS detected multiple infections of *E.coli* and CMV in patient C74. We detected *M.hominis* in patient C80. The clinician added azithromycin according to the mNGS results, and his temperature and CSF WBC/protein ratio both decreased (**Table 3**).

**Table 3:** Clinical management of 16 patients based on mNGS*.

	Culture	HSV-PCR	mNGS	Empirical antibiotics	Adjusted antibiotic	Outcome
C54	/	/	*S.epidermidis*	Ampicillin sulbactam/Ceftazidime	Vancomycin	Cured
C55	/	/	CMV	Ampicillin sulbactam/Meropenem	/	AAD
C59	/	/	*E.coli*	Meropenem	Meropenem	Cured
C62	/	/	*E.coli*	Meropenem	Ampicillin sulbactam	Cured
C63	/	HSV-2	HSV-2	Ampicillin sulbactam	Acyclovir	AAD
C65	/	/	*E.faecium*	Vancomycin	Vancomycin	Cured
C69	/	/	*B.fragilis*	Ampicillin sulbactam/Ceftazidime	Meropenem	Cured
C71	/	/	*E.faecalis*	Ampicillin sulbactam/Meropenem	Ampicillin sulbactam/Meropenem	Cured
C73	/	/	CMV	Ampicillin sulbactam	Ampicillin sulbactam/Ganciclovir	Cured
C74	/	/	*E.coli*, CMV	Ampicillin sulbactam/Meropenem	Meropenem/Ganciclovir	Cured
C76	/	/	*S.agalactiae*	Penicillin	Penicillin	Cured
C77	*E.coli*	/	*E.coli*	Ampicillin sulbactam/Meropenem	Ampicillin sulbactam/Meropenem	AAD
C80	/	/	*M.hominis*	Ampicillin sulbactam/ Ceftazidime/Vancomycin/Meropenem/Acyclovir	Azithromycin	AAD
C84	/	/	CMV	Ampicillin sulbactam/Ceftazidime	Ganciclovir	Cured
C85	/	HSV-1	HSV-1	Meropenem	Acyclovir	AAD
C88	/	/	*E.coli*	Meropenem	Meropenem	AAD

*mNGS denotes metagenomic next-generation sequencing, HSV denotes herpes simplex virus, PCR denotes polymerase-chain-reaction, and AAD denotes against advice discharge.

Among these 16 patients, three were identified by both mNGS and conventional tests, and 13 (14.8%) were identified by mNGS alone. Twelve of 13 diagnoses based solely on mNGS had a likely clinical effect (one patient’s parents stopped treatment), with nine of 13 changing antibiotic treatments. Ampicillin sulbactam was used in high doses (ampicillin,200-300mg/kg·d, q6h).

**Figure 3:**
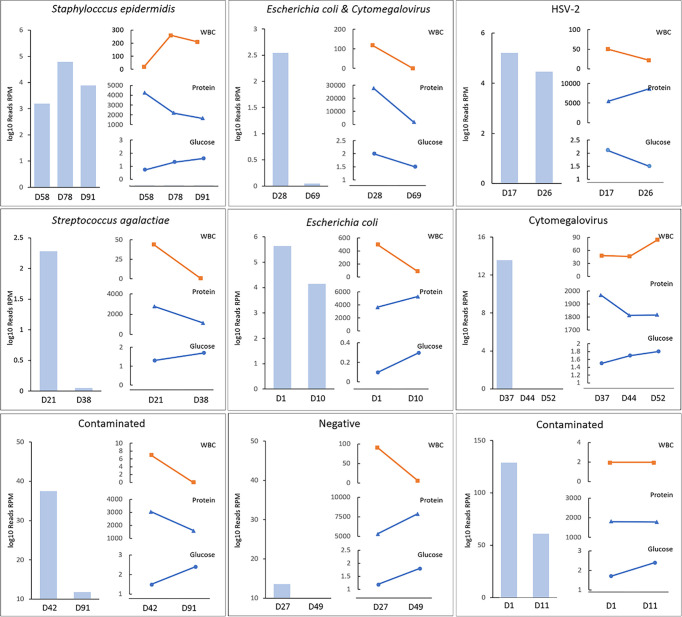
The semiquantitative value of mNGS in the dynamic surveillance of CNS infections. HSV denotes herpes simplex virus. The horizontal axis represents patients’ day of life when mNGS was conducted. Case1 was *S.epidermidis*. Case 2 was *E.coli* and cytomegalovirus. Case3 was HSV-2. Case 4 was *S.agalactiae*, Case 5 was *E.coli*, Case 6 was cytomegalovirus, Case 7 & 9 were diagnosed as contaminated although reads were detected, and Case 8 was negative. Case 8 was clearly diagnosed with GBS meningitis and treated with penicillin for 19 days in the first hospital. He was sent to our hospital after complications occurred. However, two consecutive mNGS tests were negative, and the CSF WBC count and protein and glucose levels were always abnormal.

The course of antibiotics was adjusted according to the mNGS semiquantitative sequencing reads. Due to the requirements of the diseases, nine patients underwent mNGS more than two times during their hospitalization to observe the dynamic monitoring effect of mNGS. After patients received effective antimicrobial treatment, mNGS sequencing reads decreased or even became negative, while CSF parameters (including WBC counts and protein and glucose levels) did not show a consistent correlation with the treatment (**Figure 3**). In patient C25, reads were negative two consecutive times. Although the patient still had hydrocephalus and high protein levels in the CSF, we were able to exclude infective factors and stop antibiotics in a timely manner.

## Discussion

Neonates, especially premature infants, are at higher risk of CNS infections because of the immaturity of their humoral and cellular immunity. Early diagnosis and active treatment are key to improving their prognosis. (Otto et al., 2018) mNGS has brought new perspectives to the investigation of CNS infections. We sought to explore the real-life performance of mNGS testing in a high-risk population. Previous studies have mainly described the use of mNGS in adults, but the etiology of CNS infections in neonates and adults is very different. (van de Beek et al., 2016) Our study focused on neonates and included detailed clinical data. *E. coli* remains the most common cause of bacterial meningitis in neonates, which supports previous studies reporting the epidemiology of bacterial meningitis in neonates. (Ouchenir et al., 2017) In 101 samples, mNGS showed a higher diagnostic yield than conventional methods (19.8% vs. 4.95%).

### mNGS Had Excellent Diagnostic Performance for Neonatal CNS Infections

The CSF samples were tested by using both mNGS and conventional methods. The detection rate of mNGS of 19.8% was significantly higher than that of conventional methods, while the percentage was lower than the 27.9-60% that has been reported in the literature. (Granerod et al., 2010; Wilson et al., 2019) mNGS showed a high sensitivity and specificity compared to conventional methods. Zhen Yu Li et al. recommend that mNGS be routinely applied in the diagnosis of CNS infections in immunocompromised patients, who are more susceptible to unexpected pathogens than immunocompetent patients. (Li et al., 2021) The top three causative pathogens identified were *E. coli*, *S. aureus* and CMV. Some studies have shown that group B streptococcus (GBS) is a significant pathogen responsible for neonatal CNS infections; (Romain et al., 2018) however, in this study, only one case was detected by mNGS. Four cases of GBS were detected in the first hospital and treated with penicillin. These patients were sent to our hospital after complications occurred. However, both culture and mNGS were negative, which is strongly related to the antibiotics. Newborns are people with immature immune function, so we recommend the application of mNGS to diagnose neonatal CNS infection.

We verified these positive cases by qPCR, and six of them were negative. The six patients were also diagnosed with CNS infection based on their clinical manifestations and favorable response to antibiotic. Currently, no established robust criteria reliably define a true mNGS positive result without the requirement of confirmatory testing. Alternatively, the degradation of stored pathogen DNA and/or the low abundance of pathogen DNA in the tested samples led to the failure of qPCR to replicate some of the mNGS findings. (Hong et al., 2020) CMV in the mNGS results of three patients (C55,C73, and C84) and *Enterococcus faecium* in the mNGS of patient C65 were not verified by qPCR, potentially due to the low pathogen load. However, we are currently unable to provide a good explanation for the verification result of *Bacteroides fragili* (**Supplementary Table 1**). We know that a modern and standard diagnostic microbiology extensively assesses CSF, including the use of multiple PCRs. Although the reactions are targeted, multiple PCRs provide results in as fast as 2 hours and at a much lower cost than mNGS. However, multiple PCRs of CSF are temporarily unavailable at our institution. In addition, the currently used common panels lack tools to detect *S.epidermidis* and *E. faecium*, while the infections caused by these two bacterial species were confirmed by mNGS in our study.

### mNGS Is Less Affected by Antibiotics in the Early Stage of an Infectious Disease and Is More Suitable for Neonates

The clinical manifestations of neonatal CNS infection are nonspecific, and the condition changes rapidly, prompting neonatologists to use empirical antibiotics for most patients with abnormal performance. (Mukhopadhyay et al., 2019) The application of empirical antibiotics in the neonatal population is higher than in other age groups. (McPherson et al., 2018) The application of antibiotics would affect the detection of microorganisms. In this study, mNGS showed higher detection rates than conventional methods in patients treated with or without antibiotic. mNGS detected far more potential pathogens than conventional methods, especially in the empirical treatment group. Several studies have shown that the detection rate of a CSF culture for meningitis could be reduced to 9%–11% after effective treatment. (Nigrovic et al., 2008) mNGS is more suitable than culture in neonates who are treated widely by empirical antibiotics.

### Guidance of Clinical Treatment Is the Key to Evaluating the Clinical Usefulness of mNGS

In practice, many neonates are diagnosed with clinical CNS infections based on CSF parameters and clinical manifestations. Harmony P. et al. confirmed that meningitis may occur in the presence of normal CSF WBC counts, glucose and protein levels and that no single CSF value could reliably exclude the presence of meningitis in neonates. (Garges et al., 2006) In addition, the disadvantages of the common laboratory tests in diagnosing CNS infection are low sensitivity, time-consuming. Adjusting antibiotics based on changes in WBC counts, glucose and protein level in CSF may lead to inappropriate antibiotic selection or inadequate treatment duration. (Straus et al., 2006) However, the semiquantitative reads of mNGS, which change dynamically depending on disease progression, (Ai et al., 2018) were directly correlated with effective treatment. Therefore, mNGS reads may be a better standard for evaluating the efficacy of antibiotic therapy and may guide the duration of antibiotic use. Twelve of 13 diagnoses made solely based on mNGS had a likely clinical effect, with nine of 13 guiding treatment. CMV was detected in four CSF samples, including one case of multiple infection with *E. coli.* These pathogens were only detected by mNGS. CMV infects the fetal encephalon during early gestation and compromises neurodevelopment, resulting in varying degrees of neurological damage. (Zhang and Fang, 2019) Ganciclovir therapy that begins in the neonatal period in symptomatically infected infants with CMV infection involving the central nervous system prevents hearing deterioration. (Kimberlin et al., 2015) Based on the mNGS results, we administered targeted treatments to these four patients, which substantially improved their neurological prognosis. Notably, in patient C81, *M. hominis* was detected by mNGS. Although his temperature and CSF WBC count both decreased after treatment with azithromycin, he already had severe encephalomalacia. Therefore, this result shows the importance of early accurate pathogen diagnosis and active treatment.

### The Combination of CSF mNGS and WES Is Helpful for Determining the Definitive Diagnosis of Neonates With a Suspected CNS Infection

Interestingly, we found a PID in a neonate with multiple infections by WES. Although seizures are reported to be seen in 40% of neonates with meningitis, (Heath et al., 2011) we found that the other five patients showed symptoms of suspected CNS infections, such as seizures, dystonia, or fever, and the true etiology was finally identified through WES. The value of genetic testing in newborns has been proven in some studies, e.g., early infant deaths (Yang et al., 2020) and critically ill infants. (van der Sluijs et al., 2019; Wang et al., 2020) Therefore, we also made full use of the function of NGS in this study. CSF mNGS combined with WES increased the diagnostic rate, which is more conducive to accurate treatment, provides a better understanding of the genetic basis of a rare disease and translates to more accurate prognosis, management, surveillance and genetic advice.

### Limitations

First, this study had a relatively small sample size of conventional positive results, and therefore was unable to sufficiently evaluate the diagnostic performance of mNGS in CSF. Second, mNGS has many limitations in this setting, in particular, the cost, complexity of the test, and longer turn-around than many other molecular or even culture-based tests. Furthermore, mNGS data are often difficult to interpret because NGS reads often map to multiple pathogens and because of the presence of contaminants. Moreover, we did not include a control group of patients to whom mNGS was not offered, and thus the study was limited in its ability to make conclusions about the effect of mNGS.

## Conclusions

The use of CSF mNGS has the potential to overcome several limitations of conventional CNS infectious diagnostics. mNGS showed excellent diagnostic performance for neonatal CNS infections, especially after treatment with empirical antibiotics. For patients with CNS infections or with an unclear clinical etiology, WES should be completed in a timely manner for early detection in newborns with genetic diseases and guidance from genetic consultation and clinical strategies.

## Data Availability Statement

The datasets presented in this study can be found in online repositories. The names of the repository/repositories and accession number(s) can be found below: NCBI BioProject, accession no: PRJNA66268.

## Ethics Statement

The studies involving human participants were reviewed and approved by The study was conducted in accordance with the Declaration of Helsinki. The study was approved by the Research Ethics Committee of the Children’s Hospital of Fudan University (NO.2019-300). Written informed consent to participate in this study was provided by the participants’ legal guardian/next of kin.

## Author Contributions

The data analyses were conducted by WZ and LH. Concept and design: WZ, LH, MMG, and MYG. Acquisition, analysis, or interpretation of data: MMG, MYG, LY, BW, MX, YB, RZ, and JW. Drafting of the manuscript: WZ, LH, MMG, and MYG. Critical revision of the manuscript for important intellectual content: MMG, MYG, GC, LW, YC, LH, and WZ. Statistical analysis: MMG, FX, and KY. All authors contributed to the article and approved the submitted version.

## Funding

This work was supported by the Science and Technology Commission of Shanghai Municipality (19495810300).

## Conflict of Interest

The authors declare that the research was conducted in the absence of any commercial or financial relationships that could be construed as a potential conflict of interest.
