# Supplementary material for: Combining Metagenomic Sequencing With Whole Exome Sequencing to Optimize Clinical Strategies in Neonates With a Suspected Central Nervous System Infection
Source: Front Cell Infect Microbiol. 2021 Jun 18;11:671109. doi: 10.3389/fcimb.2021.671109 (PMC8253254; doi:10.3389/fcimb.2021.671109)
Supplement: Supplementary file 1 [file DataSheet_1.docx]

**Supplementary Methods**

**CSF culture**

The CSF specimens were cultured on blood and chocolate plates (5%-10% CO2 environment), and incubated at 35°C for 48h. After incubation, positive specimens were isolated for pathogenic bacteria. Bacterial identification was performed using a VITEK2 COMPACT automated ID/AST instrument.

**Pathogen validation**

Taqman Gene Expression Assays were used to validate the pathogens detected by mNGS. For each pathogen detected by mNGS, strain name was submitted to ThermoFisher Scientific to design TaqMan assays. A sample of 5 ul DNA used for metagenomic sequencing was mixed with 2.5 ul TaqPath™ 1-Step RT-qPCR Master Mix and 2.5 ul pool of PCR preamp assays. The preamplification reaction was performed as per the manufacturer’s instructions, with up to 14 cycles. Preamplification products were diluted 1:10 in nuclease free water. A sample of 2 ul diluted products was mixed with TaqMan Fast Advanced Master Mix (10 ul), TaqMan assays (1 ul) and nuclease-free water (7 ul). Forty cycles of Real-time PCR was performed in the StepOnePlus Real-Time PCR System as per the manufacturer’s instructions.
