# Supplementary material for: Combining Metagenomic Sequencing With Whole Exome Sequencing to Optimize Clinical Strategies in Neonates With a Suspected Central Nervous System Infection
Source: Front Cell Infect Microbiol. 2021 Jun 18;11:671109. doi: 10.3389/fcimb.2021.671109 (PMC8253254; doi:10.3389/fcimb.2021.671109)
Supplement: Supplementary file 2 [file Table_1.docx]

**Supp Table 1** **qPCR validation of mNGS detected results^*^**

|  | Strain_name | Reads_num | Assay ID | qPCR validation | Crt |
| --- | --- | --- | --- | --- | --- |
| C54-T1 | *Staphylococcus epidermidis* | 614 | Ba04646141_s1 | *Staphylococcus epidermidis* | 24.39 |
| C55 | Human betaherpesvirus 5 | 29 | Vi06439643_s1 | NA | NA |
| C54-T2 | *Staphylococcus epidermidis* | 17926 | Ba04646141_s1 | *Staphylococcus epidermidis* | 14.27 |
| C54-T3 | *Staphylococcus epidermidis* | 1214 | Ba04646141_s1 | *Staphylococcus epidermidis* | 19.54 |
| C59 | *Escherichia coli* | 17 | Ba04646242_s1 | *Escherichia coli* | 26.11 |
| C62 | *Escherichia coli* | 2164 | Ba04646242_s1 | *Escherichia coli* | 23.77 |
| C63-T1 | Human alphaherpesvirus 2 | 22159 | Vi04646232_s1 | Human alphaherpesvirus 2 | 26.93 |
| C63-T2 | Human alphaherpesvirus 2 | 16282 | Vi04646232_s1 | Human alphaherpesvirus 2 | 27.45 |
| C65 | *Enterococcus faecium* | 56 | Ba04932086_s1 | NA | NA |
| C69 | *Bacteroides fragilis* | 1743 | Ba04646225_s1 | NA | NA |
| C71 | *Enterococcus faecium* | 97 | Ba04932086_s1 | *Enterococcus faecium* | 28.91 |
| C73 | Human betaherpesvirus 5 | 4 | Vi06439643_s1 | NA | NA |
| C74-T1 | *Escherichia coli* | 111 | Ba04646242_s1 | *Escherichia coli* | 28.20 |
| C74-T2 | Human betaherpesvirus 5 | 45 | Vi06439643_s1 | Cytomegalovirus | 23.77 |
| C76 | *Streptococcus agalactiae* | 37 | Ba04646276_s1 | *Streptococcus agalactiae* | 26.09 |
| C77-T1 | *Escherichia coli* | 17054260 | Ba04646242_s1 | *Escherichia coli* | 4.83 |
| C77-T2 | *Escherichia coli* | 9387 | Ba04646242_s1 | *Escherichia coli* | 22.53 |
| C80 | *Mycoplasma hominis* | 3 | Ba04646255_s1 | NA | NA |
| C84 | Human betaherpesvirus 5 | 15 | Vi06439643_s1 | NA | NA |
| C85 | Human alphaherpesvirus 1 | 916 | Vi04230116_s1 | Human alphaherpesvirus 1 | 30.32 |
| C88 | *Escherichia coli* | 216 | Ba04646242_s1 | *Escherichia coli* | NA |

*qPCR denotes Real-time Quantitative polymerase chain reaction, mNGS denotes metagenomic next-generation sequencing, HSV denotes herpes simplex virus, NA denotes negetive. T1 denotes the first test of mNGS, T2 denotes the second test of mNGS, T3 denotes the third test of mNGS.

C55. His blood CMV-PCR indicated that viral load was 1.35×10^5^, and CMV-IgM was positive. More importantly, his cranial MRI also showed typical sign of CMV infection. CMV infection was considered after consultation by the infective physician, and ganciclovir antiviral therapy was recommended.

C65. He was a 31+5w premature baby. He was diagnosed as bacterial meningitis with hydrocephalus shortly after birth, but his cerebrospinal fluid culture was negative for many times. And he was given antibiotics for 28 days before he was transferred to our hospital. To be sure, according to the results of mNGS, his hydrocephalus was effectively controlled after we applied vancomycin.

C69. He had a history of fever for 5 days. And he was given antibiotics for 1 day before the CSF was sent to mNGS. Though his CSF parameters (white blood cell, protein, glucose) were all normal, mNGS results showed *Bacteroides fragilis*, and reads were relatively high. Curiously, the validation was negative. We could not provide a good explanation for the verification result at present. According to mNGS, his body temperature returned to normal after adequate treatment with meropenem.

C73. He was diagnosed as *Streptococcus agalactiae* meningitis in the first hospital. After treatment with penicillin, cerebrospinal fluid parameters were always abnormal. In our hospital, his CSF mNGS indicated CMV, and blood CMV-IgM was positive. Ganciclovir antiviral therapy should be considered after consultation with the infective physician. CSF parameters improved after one week.

C80. Although he was treated with multiple antibiotics, his temperature and cerebrospinal fluid parameters continued to be abnormal. Until the clinician added azithromycin according to mNGS, his temperature and CSF white blood cell/protein both decreased.

C84. His blood CMV-PCR indicated that viral load was 1.82×10^4^, and CMV-IgM was positive. More importantly, his cranial MRI also showed typical sign of CMV infection, and he also had CMV infection-related fundus lesions, hearing loss and cholestasis. CMV infection was considered after consultation by the infective physician, and ganciclovir antiviral therapy was recommended.

C88. He began to develop fever seven days after birth. In the first hospital, blood and CSF cultures results suggested for *Escherichia coli.* Three days after meropenem treatment, he was transferred to our hospital. The count of white blood cells in the CSF we reviewed was still high, and mNGS also indicated that it was *Escherichia coli* whose reads were not low, but the verification was negative. The verification result exceeded our expectations, but we did not have a good explanation at present.
